# Metabolic effects of exercise on childhood obesity: a current view

**DOI:** 10.1016/j.rpped.2014.11.002

**Published:** 2015-03

**Authors:** Santiago Tavares Paes, João Carlos Bouzas Marins, Ana Eliza Andreazzi

**Affiliations:** a Universidade Federal de Juiz de Fora, Juiz de Fora, MG, Brazil; b Universidade Federal de Viçosa, Viçosa, MG, Brazil

**Keywords:** Exercise, Pediatric obesity, Child nutrition, Metabolism

## Abstract

**OBJECTIVE:**

To review the current literature concerning the effects of physical exercise on several metabolic variables related to childhood obesity.

**DATA SOURCE:**

A search was performed in Pubmed/MEDLINE and Web of Science databases. The keywords used were as follows: Obesity, Children Obesity, Childhood Obesity, Exercise and Physical Activity. The online search was based on studies published in English, from April 2010 to December 2013.

**DATA SYNTHESIS:**

Search queries returned 88,393 studies based on the aforementioned keywords; 4,561 studies were selected by crossing chosen keywords. After applying inclusion criteria, four studies were selected from 182 eligible titles. Most studies found that aerobic and resistance training improves body composition, lipid profile and metabolic and inflammatory status of obese children and adolescents; however, the magnitude of these effects is associated with the type, intensity and duration of practice.

**CONCLUSIONS:**

Regardless of the type, physical exercise promotes positive adaptations to childhood obesity, mainly acting to restore cellular and cardiovascular homeostasis, to improve body composition, and to activate metabolism; therefore, physical exercise acts as a co-factor in fighting obesity.

## Introduction

Obesity is a metabolic disorder characterized by a chronic inflammatory condition and excessive accumulation of body fat, which represents a health risk and contributes to the development of other diseases, such as type 2 diabetes, hypercholesterolemia, arterial hypertension, cardiovascular disease, obstructive sleep apnea syndrome, musculoskeletal impairments and several types of cancers.^1^^,^ 2

The etiology of obesity seems to be associated with several factors, such as genetic polymorphisms,^3^^,^ 4 dysfunction of the hypothalamic hormone signaling related to satiety, appetite and hunger,^5^^,^ 6 increased release of proinflammatory adipokines by white adipose tissue, and positive energy balance, in which the high total calorie intake, mainly high intake of energy-dense foods rich in saturated fats,^7^ sugar and salt exceeds daily calorie requirement.^8^

The development of obesity in the early stages of life is associated with the maintenance of the physiopathological state during adulthood. Childhood obesity can be defined as a condition of excessive accumulation of body fat in adipose tissue during childhood, with negative implications for health.^9^ The worldwide prevalence of childhood obesity is rapidly increasing in recent decades, being characterized as a global epidemic.^9^ In recent decades, children have become less active, probably encouraged by technological advances and socioeconomic factors.^10^ Obesity in childhood is the most important known risk factor for cardiovascular disease in adulthood, and these factors, when present in childhood, increase later in life, so it is necessary to fight them since the early stages of life, especially in relation to the life habits observed during this period.^11^

The benefits that physical exercises have on individuals' health have been well established, by improving cardiorespiratory fitness, body composition, and psychosocial well-being, among others. Physical exercise has been used as an important tool in the prevention and treatment of obesity^12^ by developing physical qualities that positively alter body composition, metabolic activity and by attenuating the comorbidities associated with excess weight.^4^^,^ 13 ^-^ 15

An inverse association has been demonstrated between physical activity level and development of obesity, mainly in the early stages of life,^9^^,^ 11 ^,^ 16 ^,^ 17 which justifies adherence to these practices, especially by children. While physical activity recommendations are well established for the adult population to fight obesity and its effects, the magnitude of the volume, intensity and frequency of physical activity is still controversial in the pediatric population.^12^

Considering that most clinical recommendations for treatment of obesity are based on the combination of several interventions, such as changing eating habits, medication use, regular physical activity and others, it is necessary to identify, assess or quantify the magnitude of the contribution of the possible types of treatment. Therefore, given the multifactorial nature of obesity, it is necessary to explain, in fact, the degree of contribution of physical exercise in the reduction and treatment of childhood obesity and its associated comorbidities. 

Thus, the aim of this study was to review the current literature regarding the effects of exercise on several metabolic variables of childhood obesity.

## Method

**Figure 1:**
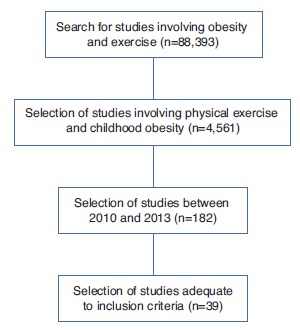
Article selection for the study.

A literature review was performed, focusing on studies that reported the effects of exercise on several metabolic variables involved in childhood obesity. The databases used for this review were PubMed/MEDLINE and Web of Science. The descriptors applied were: Obesity, Children Obesity, Childhood Obesity, Exercise and Physical Activity. The electronic search was based on studies published from April 2010 to December 2013. Therefore, we aimed to focus on the most current findings in the literature on the subject. Inclusion criteria were randomized controlled studies involving the pediatric population around 12 years old, published in English, which associated the effects of physical exercise on metabolic variables associated with childhood obesity. Initially, after a wide selection, the articles were systematically read, analyzed and listed, aiming to confront the variables of interest in the study with the literature findings. Figure 1 shows the design of the study selection.

## Results and discussion

The main identified metabolic effects of physical exercise on childhood obesity are described in Table 1.

**Table 1:** Main metabolic effects of exercise on childhood obesity.

Reference	Origin	(n) F– M	Age(yrs.)	Nutritional status	Assessed Parameters	Type of Exercise	Results
Militão et al, 2013^37^	Brazilians	34 (17-17)	9-11	Overweight and obese	Energy expenditure and health habits	Recreational activities	↓%F ↓SBP ↓TC ↓TG ↓LDL ↑VO_2max_
Laguna et al, 2013^29^	Spaniards	437 (227-210)	8-11	Obese and normal weight	HRV and Cardiometabolic risk	Cycle ergometer	Inverse association between HRV and BMI
Schranz et al, 2013^38^	Australians	56 (0-56)	13-17	Overweight and obese	Resistance Training and Body Composition	Resistance exercises	↑MM; ↓%F and ↓BMI
Lai et al, 2013^3^	Chinese	88 (48-40)	10-16	Obese	Genetic Polymorphism and Exercise	Aerobics	↓resting HR; ↓%F ↓GI ↓Dyslipidemia
Lee et al, 2012^35^	North-Americans	45 (0-45)	12-16	Obese	Metabolic effects of aerobic and resistance exercises	Aerobics and Resistance	↑MM; ↓%F; ↓BMI ↑VO_2max _; ↓BW
Davis et al, 2012^36^	North-Americans	222 (128-94)	9 -10	Overweight and obese	Dose of exercise and risk of T2DM	Aerobics	↓GI; ↓IR; ↓%F; ↓BMI
Araujo et al, 2012^32^	Brazilian	30 (21-9)	8-12	Obese	Endurance and Resistance training	Aerobics and Resistance	↑VO_2max_; ↓GI; ↓Insulinemia; ↓BMI
Park et al, 2012^28^	Koreans	29 (15-14)	11-12	Overweight and obese	Physical Activity and Endothelial Dysfunction	Aerobics and Resistance	↑VO_2max_; ↓AC ↓BMI; ↑NO; ↑Vasodilation
Makni et al, 2012^20^	Tunisians	131 (63-68)	12-14	Obese	Field Testing and lipolytic rate	Walking	Correlation VO_2max_ and %F
Legantis et al, 2012^30^	Greeks	48 (23-25)	10-11	Obese and normal weight	Cardiorespiratory Fitness and Hemodynamic Response	Isometric hand grip	↑SNA; ↑CO; ↑SBP
Woo et al, 2012^26^	Koreans	39 (19-20)	10-12	Obese and normal weight	Detraining, Adipokines and Lipid Profile	Aerobics	Negative effect of ↓LPA on lipid profile
Plonka et al, 2011^25^	Polish	59 (59-0)	9-15	Normal weight	Physical Activity Level and Leptin	Daily Energy Expenditure	Negative correlation between LPA, leptin and fat accumulation
Zorba et al, 2011^18^	Turkish	40 (0-40)	11-12	Obese	Effects of Exercise on Cardiometabolic Risk	Aerobics and Recreational activities	↓%F; ↓TC; ↓TG; ↓LDL; ↓Insulin; ↑HDL
Rosa et al, 2011^23^	North-Americans	66 (32-34)	11-14	Obese and normal weight	Physical Exercise and Inflammatory Cytokines	Aerobics with Interval	↑acute Inflammation in obese individuals
Velez et al, 2010^39^	Hispanics	28 (13-15)	15-16	Overweight and obese	Resistance Training and Body Composition	Resistance	↑MM ; ↓%F; ↓BMI

%F, Percentage of fat; SBP, systolic blood pressure; TC, Total Cholesterol; TG, Triglycerides; LDL, Low-density lipoprotein; VO_2max_, maximal oxygen uptake; HDL, high-density lipoprotein; HRV, heart rate variability; MM, muscle mass; BMI, Body Mass Index; HR, heart rate; GI, Glucose Intolerance; BW, body weight; T2DM, type 2 Diabetes Mellitus; IR, Insulin Resistance; AC, abdominal circumference; NO, Nitric Oxide; SNA, Sympathetic Nervous Activity; CO, cardiac output; LPA, level of physical activity.

## Physical exercise, metabolic rate and lipid profile

The results of this review demonstrate the effect of systematic and targeted physical exercise on metabolic variables associated with childhood obesity.^9^^,^ 10 ^,^ 16 ^-^ 18 The evidence associates the practice of exercises to body composition improvement, promoting physiological potentials that involve positive changes regarding the promotion of health and physical fitness.

The main physiological and metabolic effects resulting from both acute and chronic exercise, in general, are: increase in skeletal muscle mass, strength and proprioception gain, decrease in fat stores, increase in caloric expenditure, increased metabolic rate at rest, increased tolerance to glucose use as energy substrate, improved insulin sensitivity, decreased inflammatory state, among others.^12^^,^ 17 ^,^ 18

The increase in energy expenditure secondary to physical exercise occurs by stimulating the metabolic reactions and the enhancement of energy substrate use by active muscles. This occurs both acutely and by physiological adaptations that stimulate metabolism throughout the day.^14^ Leisure activities of moderate intensity and practiced for fun for 12 weeks were effective in attenuating dyslipidemia and hemodynamic factors associated with the worsening of the health status of obese children, with a mean body mass index (BMI) of 40 kg/m².^18^

A study carried out by Escalante et al^19^ reported that physical exercise can reduce low-density lipoproteins (LDL) by 35% and triglycerides by 40%, and increase high-density lipoproteins (HDL) in up to 25%. Therefore, exercise is considered by many authors as the main tool to attenuate the damage associated with childhood obesity.^9^^,^ 12 ^,^ 16 Makni et al^20^ evaluated the correlation between the 6-minute walk test and the use of fat as an energy substrate (FatMax) in 131 obese children (12.4±0.4 years). The study showed that the distance traveled during the test correlated significantly with the maximum heart rate achieved at the end of the walk, with this correlation being positive for boys (r=0.88) and girls (r=0.81). Thus, the researchers demonstrated that the field test is capable of quantifying the lipolytic rate of the obese child, i.e., how much the child is able to metabolize fat as an energy substrate, which makes the walk test a good clinical tool to estimate caloric expenditure. In the absence of an ergospirometer, this simple field test could be used to estimate VO_2max_ and stratify aerobic physical training loads in obese young individuals.^20^

It is noteworthy, therefore, the beneficial role of exercise in regulating the lipid profile of obese children and as an attenuator of risk factors associated with metabolic syndrome, a pathological condition that involves, in addition to the dyslipidemic and obesogenic characteristics, the presence of hypertension, insulin resistance and altered fasting glucose.^11^^,^ 16 ^,^ 17 ^,^ 18 ^,^ 21 ^,^ 22

## Physical exercise and inflammatory status

It is known that one of the characteristics of obesity is triggering a systemic inflammatory process caused by a regulatory hormonal dysfunction arising mainly from increased release of proinflammatory cytokines in the bloodstream, even during physical exercise.^23^ However, studies demonstrate that regular physical exercise is associated with the reduction of the systemic inflammatory state observed in childhood obesity.^24^

Lai et al^3^ evaluated, in 88 Chinese children, the association of the polymorphism of the recently discovered adipokine vistatin on metabolic variables. The researchers investigated the association of vistatin and the effect of an aerobic training program (20-40% of heart rate reserve), performed four times a week for four weeks. There was a marked decrease in triacylglycerol levels and insulin sensitivity in children that had the polymorphism of this pro-inflammatory adipokine. Rosa et al^24^ reported a 92% reduction in the levels of the proinflammatory adipokine interleukin-6 and oxidative metabolites of myeloperoxidase in 47 obese children undergoing intermittent training with cycling sprints at 80% VO_2max_.

Plonka et al^25^ evaluated the association between serum leptin and physical activity level in 59 obese schoolchildren. Girls that spent at least two hours daily in physical activity were considered active. It was concluded that among the active students, serum leptin was three times lower than among the sedentary ones, suggesting an improvement in sensitivity to leptin action in active girls. Corroborating the findings, Woo et al^26^ reported a significant reduction in serum leptin and increased adiponectin in obese Korean children between 10 and 12 years submitted to moderate-intensity aerobic training for 12 weeks. Moreover, even after cessation of training, serum concentrations of these adipokines remained unchanged for three subsequent months, during which the children did not engage in physical exercise.

Physical exercise has shown to be able to decrease the systemic inflammatory state, one of the physiopathological factors of obesity. The decrease in this clinical picture leads to improved function of several systems. In parallel, cellular signaling repair at the molecular level is able to act positively on cell communication and all cascades of biochemical reactions associated with metabolic systems and utilization of glucose, amino acids and fatty acids as an energy source.

## Physical exercise and cardiovascular and neural risk factors

The metabolic and hormonal dysfunction triggered by childhood obesity is associated with cardiovascular risk factors by inducing systemic changes that, later in life, can cause cardiovascular injury, whose outcomes can culminate in death. Therefore, it is necessary to encourage preventive or remedial measures to attenuate such risk factors.^27^

It has been demonstrated that regular physical exercise can promote, as early as in childhood, positive cardiovascular adaptations. Park et al^28^ evaluated the effect of an aerobic and resistance training program on endothelial function in 29 obese children for 12 weeks. Aerobic training consisted of 30 minutes of brisk walking (approximately 60% of heart rate reserve). Resistance exercise consisted in a circuit with three exercises for the upper limbs and four for the lower limbs, with 8-12 repetitions and intensity of 60% of maximum repetitions (MR). Researchers showed a two-fold higher increase in three types of endothelial progenitor cells, that is, physical training was able to stimulate an increase in endothelial vasodilator capacity, which increases blood flow to the body and decreases the strength of ventricular ejection, decreasing cardiac overload.

The heart rate recovery time after physical exercise can be used as an important tool to measure autonomic control of the heart. Thus, the magnitude of the decrease in the number of heartbeats after performing an activity, within a short time, seems to reflect an individual's level of cardiovascular fitness. However, obese individuals have an imbalance, as early as in childhood, of this involuntary control over the heart, i.e., they require more time to decrease heart rate after physical exertion. Laguna et al^29^ performed maximal exercise test on a cycle ergometer in 437 obese Spanish children, with a mean age of 9 years, and found a positive association between the time of heart rate recovery after exercise and cardiometabolic risk factors in this population, i.e., the longer it took for the heart rate to be restored to resting rate, the lower the efficiency of cardiac work.

Corroborating these findings, Legantis et al^30^ evaluated the effect of cardiorespiratory fitness and obesity on the hemodynamic response of 24 obese children, physically active and inactive, submitted to isometric handgrip exercise at 30% MR for three minutes. Inactive obese children had higher systolic blood pressure at rest and during isometric muscle contraction, when compared to active obese children. Additionally, higher levels of muscle sympathetic nerve activity, cardiac output and oxygen consumption were observed in the inactive children. Physical inactivity promotes a decrease in the individual's mechanical efficiency in the presence of a certain exertion, that is, obesity reduces the metabolic capacity to generate work and support the energy demands of physical activity. Thus, the lower the individual's aerobic efficiency in the presence of a stimulus, the less capable the individual is to endure the intensity of a task over time. These findings demonstrate that adequate physical conditioning is a good predictor of cardiovascular health, regardless of obesity, i.e., cardiorespiratory fitness may play a protective role in the heart of the obese individuals, even during childhood. 

The practice of physical exercises promotes important neural adaptations in the cardiovascular system, positively stimulating neural pathways connected to the heart muscle and endothelial smooth muscle. This has a positive effect on hemodynamic factors, such as blood pressure, heart rate and peripheral vascular resistance, which increases the strength and capacity of cardiac ejection, distribution of blood flow and thus maximizes the availability and utilization of nutrients by the skeletal muscles.

## Effect of the types of physical exercises

Increased aerobic capacity is inversely associated with fat accumulation and cardiovascular risk factors. According to a meta-analysis by Saavedra et al*,* 31 improved aerobic fitness triggers a series of physiological stimuli that enhance oxygen uptake and utilization of fatty acids as an energy source, which reduces body fat deposits, thus decreasing obesity rates.

A study by Araujo et al^32^ demonstrated an increase in VO_2max_ in 39 obese children submitted to a 12 week-training protocol, using resistance training at 80% of maximum heart rate intercalated with sprints. There was a significant increase in absolute VO_2_ (26% vs. 19%) and relative peak VO_2_ (13.1% vs. 14.6%), as well as in insulin sensitivity. However, this proposed training showed no significant reduction in body fat, or serum glucose, cholesterol, triacylglycerols and lipoproteins. However, only measurements of subcutaneous fat were performed, as visceral fat was not assessed. It is known that visceral fat is more metabolically active and associated with cardiometabolic comorbidities. In this sense, these results should be considered with caution, as most studies show positive responses related to metabolic parameters related to obesity and physical exercise, both aerobic and resistance.^31^^,^ 33

A study carried out by Ando et al^34^ showed an increased use of fat as energy substrate 24 hours after the practice of continuous or intermittent aerobic exercise. However, the magnitude of fat utilization during the 24 hours after the exercise was higher in patients submitted to continuous exercise, suggesting that the intensity, in spite of the importance of volume loads, may be a factor that modulates the level of energy expenditure. Thus, it is suggested that fractionated activities throughout the day, with higher intensity and lower volume, may result in greater impact on daily energy expenditure. 

Lee et al^35^ assessed, for a period of three months, the effects of aerobic and resistance exercise on the accumulation of abdominal, hepatic and intramyocellular fat and insulin sensitivity in 32 obese pre-adolescent boys. Both types of exercises promoted decrease in visceral and intramyocellular fat; however, only the counter-resistance exercise caused a significant increase in insulin sensitivity. The researchers attributed this increased sensitivity to the fact that resistance exercises enhance the level of localized muscle contraction and provide a further stimulus to glucose transporter proteins into the cell.

Regarding the training volume Davis et al^36^ evaluated the effect of different volumes of aerobic training in 222 overweight and obese children during 13 weeks, with five training sessions a week. The intensity of the aerobic training was 65% of VO_2max_, the volumes were 20 and 40 minutes per session and the analyzed parameters were insulin resistance and visceral fat accumulation. Both 20- and 40-minute training sessions resulted in improved insulin sensitivity and reduced visceral fat. However, the 40-minute group showed an improvement of 83% in insulin sensitivity and the same occurred for visceral fat reduction, whose decrease was 72% higher compared to the control group.

Recreational activities are also effective to promote the attenuation of risk factors of childhood obesity. Militão et al^37^ followed 34 obese schoolchildren between 9 and 11 years during the interval between classes. The study demonstrated that a 10-week program of recreational exercise combined with a program promoting healthy lifestyle habits was able to increase the values of VO_2max_, reduce LDL, triglycerides and total cholesterol as well as blood pressure levels.

As for resistance training, studies with obese children are limited due to the difficulty in quantifying training loads. However, studies that reported the effects of resistance training on metabolic variables in obese children reported positive results regarding the potential damage the disease brings to the individual.^13^ These factors are associated with the gain of fat-free mass and decrease in fat tissue, as well as the reduction of hemodynamic pressure levels and risk factors associated with the development of cardiovascular diseases.^33^

Schranz et al^38^ evaluated the effects of a 6-month resistance training program in 56 obese adolescents aged 13-17 years. In addition to the metabolic benefits brought by the practice, such as increase in muscle mass and decrease in body fat percentage, it was observed that this type of intervention also promotes benefits related to the self-esteem of obese individuals, a factor that is indirectly associated with the psychosocial aspect related to obesity.^38^^,^ 39

Similarly, it was demonstrated that resistance training promotes many metabolic benefits, such as improved insulin sensitivity, increased glucose utilization as energy substrate and improved lipid profile, factors closely associated with childhood obesity impairment.^13^

Resistance exercises performed in the traditional way, such as in fitness centers, are usually not well accepted by the pediatric population. Thus, it is important that recreational activities such as games and/or sports that involve the body's own resistance or the peers' are encouraged. Sports practices that include gymnastics or combat sports in general, with special emphasis on judo, are interesting ways of working the force component in this population, mainly by stimulating the practice of pleasurable activities that require anaerobic and neuromuscular power.

Exercises with predominant aerobic characteristics should also be performed. Unlike adults, who can bear periods of continuous exercise on a cycle ergometer or running, children do not tolerate well this type of prescription. Because of this, it is interesting to encourage the practice of recreational activities and sports that involve a large amount of body mass such as soccer, futsal, basketball, handball and water polo. Swimming and activities with roller skates usually represent well-tolerated activities, which are also interesting to increase energy expenditure and improve aerobic capacity.

Thus, alternating different types of physical activity throughout the week, totaling four to six days, would have a fundamental role in daily energy expenditure, constituting a strategy to fight, prevent or mitigate the deleterious effects of childhood obesity.

## Conclusion

The practice of physical exercises has shown to promote positive adaptations on childhood obesity and act as adjuvant for its prevention and treatment. The magnitude of benefits may vary with the exercise. The main effects arising from the exercises are mainly related to the restoration of the lipid profile, autonomic and hemodynamic restoration, improved body composition, use of energy substrates and metabolic activation.
