# Supplementary material for: Metabolic effects of exercise on childhood obesity: a current view
Source: Rev Paul Pediatr. 2015 Mar;33(1):122–9. doi: 10.1016/j.rpped.2014.11.002 (PMC4436964; doi:10.1016/j.rpped.2014.11.002)
Supplement: Translation

# Translation: Efeitos metabólicos do exercício físico na obesidade infantil: uma visão atual

**Authors:** Santiago Tavares Paes, João Carlos Bouzas Marins, Ana Eliza Andreazzi

**Affiliations:** a Universidade Federal de Juiz de Fora (UFJF), Juiz de Fora, MG, Brasil; b Universidade Federal de Viçosa (UFV), Viçosa, MG, Brasil

**Keywords:** Exercício físico, Obesidade pediátrica, Nutrição da criança, Metabolismo

## Abstract

**OBJETIVO:**

Revisar a literatura atual a respeito dos efeitos do exercício físico sobre diferentes variáveis metabólicas da obesidade infantil.

**FONTES DE DADOS:**

A pesquisa foi feita nas bases de dados Pubmed e Web of Science. Os descritores usados foram: obesity, children obesity, childhood obesity, exercise e physical activity. A pesquisa eletrônica foi feita com base nos estudos publicados de abril de 2010 a dezembro de 2013, em idioma inglês.

**SÍNTESE DOS DADOS:**

O rastreamento dos estudos com os descritores encontrou 88.393. Após cruzamento entre os descritores, obtiveram-se 4.561. Desses, depois da análise dos títulos, foram cogitados 182 relevantes referências, submetidos então aos critérios de inclusão/exclusão, e totalizaram, no fim, 39. A maioria dos estudos relacionou a prática de exercícios físicos aeróbicos e resistidos à melhoria da composição corporal, à regulação do perfil lipídico e metabólico e ao estado inflamatório de crianças e adolescentes obesos. Entretanto, a magnitude dos efeitos está associada ao tipo, à intensidade e à duração da prática.

**CONCLUSÕES:**

O exercício físico, independentemente do tipo, mostra-se capaz de promover adaptações positivas sobre a obesidade infantil, principalmente por atuar na restauração da homeostase celular e sistema cardiovascular, na melhoria da composição corporal e também aumento da ativação metabólica.

## Introdução

A obesidade é um distúrbio metabólico caracterizado por um estado inflamatório crônico e acúmulo excessivo de gordura corporal, que apresenta um risco para a saúde e contribui para o desenvolvimento de outras patologias, como *diabetes melittus* tipo 2, hipercolesterolemia, hipertensão arterial, doenças cardiovasculares, síndrome de apneia obstrutiva do sono, comprometimentos osteomioarticulares e diversos tipos de cânceres. 1 ^and^ 2

A etiologia da obesidade parece estar vinculada a inúmeros fatores, tais como polimorfismos gênicos,^3^ ^and^ 4 disfunções da sinalização de hormônios hipotalâmicos vinculados à saciedade, apetite e fome,^5^ ^and^ 6 aumento da liberação de adipocinas pró-inflamatórias pelo tecido adiposo branco e balanço energético positivo, no qual a alta ingestão calórica total, em especial o consumo elevado de alimentos energéticos, ricos em gorduras saturadas,^7^ açúcares e sal, ultrapassa a necessidade calórica diária.^8^

O desenvolvimento da obesidade em estágios iniciais da vida está associado à manutenção do estado fisiopatológico durante a vida adulta. A obesidade infantil pode ser definida como um quadro de acúmulo excessivo de gordura corporal no tecido adiposo durante a infância, com implicações negativas para a saúde.^9^ A prevalência mundial da obesidade infantil vem apresentando um rápido aumento nas últimas décadas e é caracterizada como epidemia mundial.^9^ Nas últimas décadas, as crianças tornaram-se menos ativas, provavelmente incentivadas pelos avanços tecnológicos e fatores socioeconômicos.^10^ A obesidade na infância é o mais importante fator de risco conhecido para as doenças cardiovasculares na vida adulta e esses fatores, apresentados na infância, se ampliam posteriormente. Por isso, é necessário combatê-los desde fases iniciais da vida, especialmente em relação a hábitos assumidos nesse período.^11^

Estão comprovados os benefícios que o exercício físico promove para a saúde das pessoas, por atuar na melhoria da aptidão cardiorrespiratória, da composição corporal e do bem-estar psicossocial, entre outros. O exercício físico tem sido usado como importante ferramenta na prevenção e no tratamento da obesidade^12^ por desenvolver qualidades físicas que modificam positivamente a composição corporal e a atividade metabólica e por atenuar as comorbidades associadas ao excesso de peso.^4^^,^ 13 ^,^ 14 ^and^ 15

Demonstra-se associação inversa entre nível de atividade física e desenvolvimento da obesidade, principalmente em estágios iniciais da vida,^9^^,^ 11 ^,^ 16 ^and^ 17 o que justifica a adesão a essas práticas especialmente em crianças. Enquanto, para a população adulta, estão bem estabelecidas as recomendações da prática de atividade física no combate à obesidade e aos seus efeitos, para a população pediátrica, a magnitude do volume, da intensidade e da frequência de atividade ainda é controversa.^12^

Visto que a maioria das recomendações clínicas de tratamento da obesidade se baseia na união de diversas intervenções como mudança de hábitos alimentares, uso de medicamentos, prática de regular atividade física e outras, é necessário identificar, avaliar ou quantificar a magnitude da contribuição das possíveis formas de tratamento. Portanto, dado o caráter multifatorial da obesidade, é preciso explicitar, de fato, o grau de contribuição do exercício físico na atenuação e no tratamento da obesidade infantil e das comorbidades a ela associada.

Assim, o objetivo do presente estudo foi revisar a literatura atual em relação aos efeitos do exercício físico nas diferentes variáveis metabólicas da obesidade infantil.

## Método

Foi feita uma revisão da literatura com foco nos estudos que relataram os efeitos do exercício físico sobre diferentes variáveis metabólicas envolvidas na obesidade infantil. As bases de dados usadas foram Pubmed/MedLine e Web of Science. Os descritores aplicados foram: obesity, children obesity, childhood obesity, exercise e physical activity. A pesquisa eletrônica foi baseada nos estudos publicados de abril de 2010 até dezembro de 2013. Dessa maneira, buscou-se focalizar os achados mais atuais da literatura sobre o tema. Foram considerados critérios de inclusão estudos randomizados e controlados que envolveram população pediátrica em torno de 12 anos, publicados em inglês, que relacionaram os efeitos do exercício físico sobre variáveis metabólicas associadas à obesidade infantil. Inicialmente, após uma ampla seleção, os artigos foram sistematicamente lidos, analisados e relacionados com intuito de confrontar as variáveis de interesse do estudo com os achados da literatura. A figura 1 demonstra o delineamento da seleção dos estudos.

**Figura 1:** Seleção dos artigos do estudo.

## Resultados e discussão

Os principais efeitos metabólicos identificados do exercício físico sobre a obesidade infantil são descritos na tabela 1.

**Tabela 1:** Principais efeitos metabólicos do exercício físico sobre a obesidade infantil

Referência	Origem	(n) F-M	Idade (anos)	Estado nutricional	Parâmetros avaliados	Tipo de exercício	Resultados
Militão et al., 2013^37^	Brasileiros	34 (17-17)	9-11	Sobrepesos e obesos	Dispêndio energético e Hábitos de saúde	Atividades recreacionais	↓% G ↓ PAS ↓CT ↓TG ↓LDL↑VO2_max_
Laguna et al., 2013^29^	Espanhóis	437 (227-210)	8-11	Sobrepesos e eutróficos	VFC e risco cardiometabólico	cicloergômetro	Inversa associação entre VFC e IMC
Schranz et al., 2013^38^	Australianos	56 (0-56)	13-17	Sobrepesos e obesos	Treinamento de resistência e composição corporal	Resistido	↑MM; ↓% G e↓IMC
Lai et al., 2013^3^	Chineses	88 (48-40)	10-16	Obesos	PolimorfismoGenético e exercício	Aeróbico	↓FCRepouso;↓%G ↓IG ↓Dislipdemia
Lee et al., 2012^35^	Americanos	45 (0-45)	12-16	Obesos	Efeitos metabólicos do exercício físico do tipo aeróbico e resistido	Aeróbico e resistido	↑MM; ↓% G;↓IMC ↑VO2_max_ ; ↓PC
Davis et al., 2012^36^	Americanos	222 (128-94)	9 -10	Sobrepesos e obesos	Dose de exercício físico e risco de DMT2	Aeróbico	↓IG; ↓RI; ↓%G; ↓IMC
Araujo et al., 2012^32^	Brasileiros	30 (21-9)	8-12	Obesos	Treinamento de resistência e resistido	Aeróbico e resistência	↑VO2_max_ ; ↓IG; ↓Insulinemia; ↓IMC
Park et al., 2012^28^	Coreanos	29 (15-14)	11-12	Sobrepesos e obesos	Atividade física e disfunção endotelial	Aeróbico e resistência	↑VO2_max_ ; ↓CA; ↓IMC; ↑NO; ↑Vasodilatação
Makni et al., 2012^20^	Tunisianos	131 (63-68)	12-14	Obesos	Teste de campo e taxa lipolítica	Caminhada	Correlação VO2_max_ e % G
Legantis et al., 2012^30^	Gregos	48 (23-25)	10-11	Obesos e Eutróficos	Aptidão cardiorrespira-tória e resposta hemodinâmica	Preensão isométrica Manual	↑ANS; ↑DC; ↑PAS
Woo et al., 2012^26^	Coreanos	39 (19-20)	10-12	Obesos e eutróficos	Destreino, adipocinas e perfil lipídico	Aeróbico	Efeito negativo da ↓ NAF sobre perfil lipídico
Plonka et al., 2011^25^	Polonesas	59 (59-0)	9-15	Eutróficas	Nível de atividade física e leptina	Gasto energético diário	Correlação negativa entre NAF, leptina e acúmulo de gordura
Zorba et al., 2011^18^	Turcos	40 (0-40)	11-12	Obesos	Efeitos do exercício físico sobre risco cardiometabó-lico	Aeróbico e atividades recreacionais	↓% G; ↓CT; ↓TG; ↓LDL; ↓Insulina; ↑HDL
Rosa et al., 2011^23^	Americanos	66 (32-34)	11-14	Obesos e eutróficos	Exercício físico e citocinas inflamatórias	Aeróbico intervalado	↑ Inflamação aguda nos obesos
Velez et al, 2010^39^	Hispânicos	28 (13-15)	15-16	Sobrepesos e obesos	Treinamento de resistência e composição corporal	Resistido	↑MM; % G; ↓IMC

%G, percentual de gordura; PAS, pressão arterial sistólica; CT, colesterol total; TG, triglicérides; LDL, lipoproteína de baixa densidade; VO _max_, volume máximo de oxigênio; HDL, lipoproteína de alta densidade; VFC, variabilidade da frequência cardíaca; MM, massa muscular; IMC, índice de massa corporal; FC, frequência cardíaca; IG, intolerância a glicose; PC, peso corporal; DMT2, *diabetes mellitus* do tipo 2; RI, resistência a insulina; CA, circunferência abdominal; NO, óxido nítrico; ANS, atividade nervosa simpática; DC, débito cardíaco; NAF, nível de atividade física.

### Exercício físico, taxa metabólica e perfil lipídico

Os resultados desta revisão evidenciam o poder que a prática sistematizada e orientada do exercício físico desempenha sobre variáveis metabólicas associadas à obesidade infantil.^9^^,^ 10 ^,^ 16 ^,^ 17 ^and^ 18 As evidências associam a prática de exercícios à melhoria da composição corporal e à promoção de potencialidades fisiológicas que envolvem modificações positivas no que diz respeito à promoção de saúde e ao condicionamento físico.

Os principais efeitos fisiológicos e metabólicos proporcionados pelo exercício físico, tanto agudo quanto crônico, de uma maneira geral são: aumento da massa muscular esquelética, ganho de força, propriocepção, diminuição dos estoques de gordura, aumento do gasto calórico, aumento da taxa metabólica de repouso, aumento da tolerância ao uso da glicose como substrato energético, melhoria da sensibilidade insulínica, diminuição do estado inflamatório, entre outros.^12^^,^ 17 ^and^ 18

O aumento do gasto energético secundário ao exercício físico acontece pelo estímulo das reações metabólicas e pela potencialização do uso de substratos energéticos pela musculatura ativa. Isso ocorre tanto de forma aguda quanto por adaptações fisiológicas que estimulam o metabolismo ao longo do dia.^14^ Atividades de lazer de moderada intensidade e praticadas com caráter lúdico por 12 semanas se mostraram eficientes na atenuação da dislipidemia e de fatores hemodinâmicos associados à pioria do estado de saúde de crianças obesas, com média de índice de massa corporal (IMC) de 40 kg/m^2^.^18^

Levantamento feito por Escalante et al.^19^ relatou que o exercício físico é capaz de reduzir as lipoproteínas de baixa densidade (LDL) em até 35% e os triacilgliceróis em 40%, além de aumentar as lipoproteínas de alta densidade (HDL) em até 25%. Dessa maneira, o exercício físico é considerado por diversos autores como a principal ferramenta para atenuar os danos associados à obesidade infantil.^9^^,^ 12 ^and^ 16 Makni et al.^20^ avaliaram a correlação entre o teste de caminhada de seis minutos e o uso de gordura como substrato energético (FatMax) em 131 crianças obesas (12,4 ± 0,4 anos). O estudo demonstrou que a distância percorrida durante o teste está significativamente correlacionada à frequência cardíaca máxima alcançada no fim da caminhada e tal correlação é positiva para meninos (r = 0,88) e meninas (r = 0,81). Dessa maneira, os pesquisadores demonstraram que o teste de campo é capaz de quantificar a taxa lipolítica da criança obesa, ou seja, o quanto ela é capaz de metabolizar a gordura como substrato energético, o que faz do teste de caminhada uma boa ferramenta clínica para a estimativa de gasto calórico. Na ausência de ergoespirômetro, esse simples teste de campo poderia ser usado para estimar o VO_2máx_ e estratificar as cargas de treinamento físico aeróbico em jovens obesos.^20^

Destaca-se, portanto, o papel benéfico do exercício físico na regulação do perfil lipídico de crianças obesas e como atenuador dos fatores de risco associados à síndrome metabólica, estado patológico que envolve, além das características dislipidêmica e obesogênica, hipertensão arterial sistêmica, resistência insulínica e glicemia alterada de jejum.^11^^,^ 16 ^,^ 17 ^,^ 18 ^,^ 21 ^and^ 22

### Exercício físico e estado inflamatório

Sabe-se que uma das características da obesidade é desencadear um processo inflamatório sistêmico ocasionado por disfunção regulatória hormonal advinda principalmente do aumento da liberação de citocinas pró-inflamatórias na corrente sanguínea, mesmo durante o exercício físico.^23^ Todavia, os estudos demonstram que a prática regular de exercícios físicos está relacionada à redução do estado inflamatório sistêmico presente na obesidade infantil.^24^

Lai et al.^3^ avaliaram, em 88 crianças chinesas, a relação do polimorfismo da adipocina vistatina, recentemente descoberta, sobre variáveis metabólicas. Os pesquisadores investigaram a relação da vistatina e o efeito de um programa de treinamento aeróbico (20-40% da frequência cardíaca de reserva), feito quatro vezes por semana durante quatro semanas. Os resultados demostraram uma diminuição mais acentuada dos níveis de triacilgliceróis e sensibilidade insulínica das crianças que apresentavam o polimorfismo dessa adipocina pró-inflamatória. Rosa et al.^24^ relataram redução de 92% da concentração de adipocina pró-inflamatória interleucina-6 e metabólitos oxidativos da mieloperoxidase em 47 crianças obesas submetidas a treinamento intermitente com sprints de ciclismo à 80% VO_2max_.

Estudo de Plonka et al.^25^ avaliou a relação entre leptina sérica e nível de atividade física em 59 escolares obesas. Consideraram-se como ativas as meninas que gastavam no mínimo duas horas do dia em atividades físicas. Concluiu-se que, entre as estudantes ativas, a concentração de leptina sérica era três vezes menor do que entre as inativas, o que sugere uma melhoria da sensibilidade à ação da leptina das meninas ativas. Corroborando os achados, Woo et al.^26^ relataram redução significativa da concentração de leptina sérica e aumento de adiponectina em crianças coreanas obesas entre 10 e 12 anos submetidas a treinamento aeróbico de moderada intensidade por 12 semanas. Ademais, mesmo após a interrupção do treinamento, a concentração sérica dessas adipocinas permaneceu inalterada pelos três meses subsequentes, período em que as crianças não fizeram exercícios físicos.

O exercício físico mostra-se capaz de diminuir o estado inflamatório sistêmico, um dos fatores fisiopatológicos da obesidade. A diminuição desse quadro acarreta a melhoria da função de diversos sistemas. Paralelamente, a restauração da sinalização celular em nível molecular é capaz de agir positivamente sobre a comunicação celular e todas as cascatas de reações bioquímicas vinculadas aos sistemas metabólicos e ao uso de glicose, aminoácidos e ácidos graxos como fonte energética.

### Exercício físico e fatores de risco cardiovascular e neural

A disfunção metabólica e hormonal desencadeada pela obesidade infantil está associada a fatores de risco cardiovascular por induzir alterações sistêmicas que, no decorrer da vida, podem gerar lesões a nível cardiovascular, cujos desfechos podem culminar em óbito. Portanto, é preciso incentivar medidas preventivas ou paliativas que visem a atenuar tais fatores de risco.^27^

Demonstra-se que a prática regular de exercícios físicos é capaz de promover, já na infância, adaptações cardiovasculares positivas. Park et al.^28^ avaliaram o efeito de um programa de treinamento aeróbico e resistido sobre a função de células endoteliais em 29 crianças obesas durante 12 semanas. O treinamento aeróbico era constituído de 30 minutos de caminhada rápida (aproximadamente 60% da frequência cardíaca de reserva). O exercício resistido consistia na realização de um circuito com três exercícios de contra-resistência para membros superiores e quatro para membros inferiores, constituídos de 8-12 repetições e intensidade de 60% da máxima repetição voluntária (RM). Os pesquisadores evidenciaram um aumento duas vezes maior em três tipos de células progenitoras endoteliais, ou seja, o treinamento físico foi capaz de estimular um aumento da capacidade vasodilatadora endotelial, o que gera impacto positivo sobre o fluxo sanguíneo para o corpo, diminui a força de ejeção ventricular e diminui a sobrecarga cardíaca.

O tempo de recuperação da frequência cardíaca pós-exercício físico pode ser usado como importante ferramenta para mensurar o controle, em nível autonômico, do coração. Dessa maneira, a magnitude do decréscimo do número de batimentos cardíacos após uma atividade, dentro de um curto tempo, parece refletir o nível de aptidão cardiovascular de um indivíduo. Todavia, pessoas obesas apresentam já na infância um desbalanço desse controle involuntário sobre o coração, ou seja, necessitam de maior tempo de descanso para restaurar a frequência cardíaca de repouso após o esforço físico. Laguna et al.^29^ fizeram testes de esforço máximo em cicloergômetro em 437 crianças espanholas obesas, com idade média de nove anos, e verificaram uma relação positiva entre o tempo de recuperação da frequência cardíaca de esforço com fatores de risco cardiometabólico nessa população, ou seja, quanto maior o tempo de restauração dos batimentos cardíacos máximos até o repouso, menor a eficiência do trabalho cardíaco.

Corroborando esses achados, Legantis et al.^30^ avaliaram o efeito da aptidão cardiorrespiratória e da obesidade na resposta hemodinâmica de 24 crianças obesas, fisicamente ativas e inativas, submetidas a exercício isométrico de preensão manual a 30% da RM durante três minutos. As crianças obesas inativas apresentaram maiores valores de pressão arterial sistólica no repouso e durante a contração muscular isométrica, em comparação com as crianças obesas ativas. Adicionalmente, foram observados valores mais elevados de atividade nervosa simpática muscular, débito cardíaco e consumo de oxigênio entre as inativas. A inatividade física promove no indivíduo uma diminuição da eficiência mecânica frente a um determinado esforço, ou seja, a obesidade diminui a capacidade metabólica de gerar trabalho muscular e suportar a demanda energética da atividade física. Assim, quanto menor for a eficiência aeróbica do indivíduo frente a um estímulo, menor será a capacidade de suportar, ao longo do tempo, a intensidade de uma tarefa. Esses achados demonstram que o condicionamento físico adequado é um bom preditor de saúde cardiovascular, independentemente da obesidade, ou seja, a aptidão cardiorrespiratória pode desempenhar um papel de proteção ao coração do indivíduo obeso, mesmo durante a infância.

A prática de exercícios físicos promove importantes adaptações neurais sobre o sistema cardiovascular, estimula positivamente vias neurais ligadas ao músculo cardíaco e à musculatura lisa endotelial. Isso repercute positivamente em fatores hemodinâmicos, como pressão arterial, frequência cardíaca e resistência vascular periférica, o que aumenta a força e a capacidade de ejeção cardíaca e a distribuição do fluxo sanguíneo e, consequentemente, maximiza a disponibilidade e o uso de nutrientes pela musculatura esquelética.

### Efeito dos tipos de exercício físico

O aumento da capacidade aeróbica está associado inversamente ao acúmulo de gordura e a fatores de risco cardiovascular. De acordo com metanálise feita por Saavedra et al,^31^ a melhoria do condicionamento aeróbico desencadeia uma série de estímulos fisiológicos que potencializam a captação de oxigênio e o uso dos ácidos graxos como fonte de energia, o que reduz os depósitos de gordura corporal e diminui os índices de obesidade.

Estudo de Araujo et al.^32^ demonstrou aumento do VO_2máx_ em 39 crianças obesas submetidas a um protocolo de treinamento de 12 semanas, com treino de resistência aeróbica a 80% da frequência cardíaca pico e treinamento intervalado com *sprints*. Houve aumento significativo no VO_2_ absoluto (26% *vs.* 19%) e VO_2_ pico relativo (13,1% *vs.* 14,6%), bem como na sensibilidade insulínica. Entretanto, essa proposta de treinamento não demonstrou redução significativa de gordura corporal, tampouco dos valores séricos de glicemia, colesterol, triacilgliceróis e lipoproteínas. Todavia, apenas foi mensurada a gordura subcutânea, não foram verificados os depósitos viscerais. Sabe-se que a gordura visceral é a metabolicamente mais ativa e relacionada às morbidades cardiometabólicas. Nesse sentido, esses resultados devem ser considerados com cautela, pois grande parte dos estudos demonstra respostas positivas relacionadas aos parâmetros metabólicos vinculados à obesidade e a prática exercício físico tanto do tipo resistido quanto aeróbico. 31 ^and^ 33

Estudo desenvolvido por Ando et al.^34^ evidenciou um aumento do uso de gordura como substrato energético 24 horas após a prática de exercício aeróbico contínuo ou intermitente. Entretanto, a magnitude do uso de gordura nas 24 horas subsequentes à prática foi maior nos indivíduos submetidos ao treinamento intermitente, o que sugere que a intensidade, apesar da relevância das cargas de volume, pode ser um importante fator que modula o grau de dispêndio energético. Dessa maneira, sugere-se que atividades fracionadas ao longo do dia, com maior intensidade e menor volume, possam causar maior impacto sobre o gasto energético diário.

Lee et al.^35^ acompanharam por três meses os efeitos de exercícios físicos do tipo aeróbico e resistido em 32 meninos pré-adolescentes obesos sobre o acúmulo de gordura abdominal, hepática, intramiocelular e sensibilidade insulínica. Ambos os exercícios promoveram redução dos níveis de gordura visceral e intramiocelular, entretanto somente o exercício de contra-resistência promoveu aumento significativo da sensibilidade insulínica. Os pesquisadores atribuíram o aumento da melhoria dessa sensibilidade ao fato de o exercício resistido potencializar o nível de contração muscular localizado e promover um maior estímulo de proteínas translocadoras de glicose para dentro da célula.

Em relação ao volume de treinamento, Davis et al.^36^ avaliaram a ação de diferentes volumes de treinamento aeróbico em 222 crianças portadoras de sobrepeso e obesidade ao longo de 13 semanas, com cinco sessões de treino por semana. A intensidade do treinamento aeróbico foi de 65% do VO_2max_, os volumes foram de 20 e 40 minutos por sessão e os parâmetros analisados foram resistência insulínica e acúmulo de gordura visceral. Tanto 20 quanto 40 minutos de duração da sessão de treino proporcionaram melhoria na sensibilidade insulínica e redução de gordura visceral. Todavia, o grupo de 40 minutos apresentou uma melhoria de 83% da sensibilidade insulínica. O mesmo ocorreu para a redução de gordura visceral, cuja redução foi 72% superior ao grupo controle.

Atividades recreativas são também eficientes para promover a atenuação dos fatores de risco da obesidade infantil. Militão et al.^37^ acompanharam 34 escolares obesos entre 9 e 11 anos, durante o período de intervalo entre aulas. O estudo demonstrou que um programa de 10 semanas de exercício recreativos aliado a um programa de orientação de hábitos de vida saudáveis foi capaz de aumentar os valores de o VO2_max_ e reduzir os valores de LDL, triglicérides, colesterol total e pressão arterial.

Quanto ao treinamento resistido, os estudos com crianças obesas são limitados, devido à dificuldade de quantificar cargas de treinamento. Entretanto, pesquisas que relacionaram os efeitos do treinamento resistido sobre variáveis metabólicas em crianças obesas relatam resultados positivos no que tange aos possíveis danos que a doença exerce sobre o indivíduo.^13^ Esses fatores estão associados ao ganho de massa isenta de gordura e à diminuição do tecido adiposo, bem como à diminuição dos níveis tensionais hemodinâmicos e dos fatores de risco associados ao desenvolvimento de doenças cardiovasculares.^33^

Schranz et al.^38^ avaliaram os efeitos de um programa de seis meses de treinamento resistido em 56 adolescentes obesos, entre 13-17 anos. Adicionalmente aos benefícios metabólicos proporcionados pelo período de prática, como o aumento da massa muscular e a redução do percentual de gordura, foi observado que esse tipo de intervenção também promove benefícios relacionados à autoestima de jovens obesos, fator que indiretamente está associado ao aspecto psicossocial ligado à obesidade.^38^ ^and^ 39

De maneira semelhante, foi demonstrado que o treinamento resistido promove diversos benefícios metabólicos, como a melhoria da sensibilidade insulínica, o aumento do uso da glicose como substrato energético e a melhoria do perfil lipídico, fatores intimamente associados ao comprometimento da obesidade infantil.^13^

Exercícios resistidos feitos de forma tradicional, como academias de ginástica, normalmente não são bem aceitos pela população pediátrica. Assim, é importante que atividades lúdicas, como jogos e/ou brincadeiras, que envolvam a resistência do próprio corpo ou de companheiros, sejam estimuladas. A iniciação esportiva na ginástica ou em lutas em geral, com especial destaque para o judô, é uma forma interessante de trabalhar o componente da força nessa população, principalmente por dinamizar a prática prazerosa de atividades que requeiram potência anaeróbica e neuromuscular.

Exercícios com características eminentemente aeróbicas também devem ser realizados. Diferentemente de adultos, que suportam períodos de exercício contínuo em cicloergômetro ou corrida, as crianças não toleram bem essa forma de prescrição. Por conta disso, é interessante o incentivo e a prática de atividades recreativas e jogos esportivos que envolvam uma grande quantidade de massa corporal, como futebol, futsal, basquetebol, handebol e polo aquático. A natação e esportes com patins representam atividades normalmente bem toleradas e que também são interessantes para aumentar o gasto energético e aprimorar a capacidade aeróbica.

Dessa forma, uma alternância de atividades físicas ao longo da semana, entre quatro e seis dias, exerceria papel fundamental no dispêndio energético diário e se constituiria como estratégia para combater, prevenir ou atenuar os efeitos deletérios da obesidade infantil.

## Conclusão

A prática de exercícios físicos se mostra capaz de promover adaptações positivas sobre a obesidade infantil e atuar como coadjuvante na sua prevenção e tratamento. A magnitude dos benefícios pode variar conforme o exercício físico. Os principais efeitos oriundos dos exercícios estão vinculados principalmente à restauração do perfil lipídico, restauração hemodinâmica e autonômica, melhoria da composição corporal, mobilização dos substratos energéticos e à ativação metabólica.
